# Missed opportunities for early HIV diagnosis in Greece: The MORFEAS study, 2019 to 2021

**DOI:** 10.2807/1560-7917.ES.2024.29.48.2400138

**Published:** 2024-11-28

**Authors:** Sotirios Roussos, Nikos Pantazis, Konstantinos Protopapas, Anastasia Antoniadou, Antonios Papadopoulos, Giota Lourida, Vasileios Papastamopoulos, Maria Chini, Konstantinos Alexakis, Emmanouil Barbounakis, Diamantis Kofteridis, Lydia Leonidou, Markos Marangos, Vasileios Petrakis, Periklis Panagopoulos, Elpida Mastrogianni, Dimitrios Basoulis, Panagiota Palla, Nikolaos Sipsas, Varvara Vasalou, Vasileios Paparizos, Simeon Metallidis, Theofilos Chrysanthidis, Ioannis Katsarolis, Vana Sypsa, Mina Psichogiou

**Affiliations:** 1 Department of Hygiene, Epidemiology and Medical Statistics, Medical School, National and Kapodistrian University of Athens, Athens, Greece; 2 4th Department of Internal Medicine and HIV/ID Unit, ATTIKON University General Hospital, Athens, Greece; 3 5th Department of Internal Medicine and HIV/ID Unit, Evangelismos General Hospital, Athens, Greece; 4 3rd Department of Internal Medicine and HIV/ID Unit, ‘Red Cross’ Korgialeneio-Benakeio General Hospital, Athens, Greece; 5 Department of Internal Medicine and HIV/ID Unit, Herakleion University General Hospital (PAGNH), Herakleion, Crete, Greece; 6 Department of Medicine and HIV/ID Unit, Patras University General Hospital, Patras, Greece; 7 Department of Internal Medicine and HIV/ID Unit, Alexandroupolis University General Hospital, Alexandroupolis, Greece; 8 1st Department of Internal Medicine, Laiko General Hospital, Athens, Greece; 9 Pathophysiology Department, Laiko General Hospital, Athens, Greece; 10 1st Department of Dermatology and Venereology, HIV Unit, Andreas Syggros University Hospital, Athens, Greece; 11 1st Internal Medicine Department, Infectious Diseases Division, AHEPA Hospital, Thessaloniki, Greece; 12 Medical Affairs, Gilead Sciences Hellas and Cyprus, Paleo Faliro, Greece

**Keywords:** Missed opportunities, HIV, early diagnosis, indicator conditions, Greece, MORFEAS

## Abstract

**Background:**

Late HIV diagnosis (CD4^+^ T-cell count < 350 cells/μL, or with an AIDS-defining event) remains a persistent challenge in Greece, indicating potential missed opportunities (MOs) for earlier testing.

**Aim:**

To determine the frequency of HIV indicator conditions (ICs) preceding diagnosis and to quantify MOs for earlier testing at a nationwide level in Greece.

**Methods:**

This multicentre retrospective study analysed data on 823 antiretroviral therapy-naive adults (≥ 18 years) diagnosed with HIV during 2019–21. Medical records were reviewed to identify pre-diagnosis healthcare contacts (HCCs) and ICs justifying HIV testing. Univariable and multivariable logistic regression identified factors associated with ≥ 1 MO. A Bayesian model estimated the time from seroconversion to diagnosis.

**Results:**

Among 517 participants with HCC data, 249 had ≥ 1 HCC. Of these, 59.0% (147/249) were late presenters. These cases had 365 HCCs, and 191 (52.3%) were MOs for testing. The most common ICs were sexually transmitted infections (39.8%; 76/191) and fever (11.0%; 21/191). Non-Greek origin was associated with lower odds of experiencing ≥ 1 MO (adjusted odds ratio: 0.48; 95% CI: 0.22─1.02), while higher education increased odds of MOs for early HIV diagnosis. Median time from seroconversion to diagnosis was 3.2 years for the full sample and 3.7 years for those with HCC, with about half of the latter reporting MOs post-estimated seroconversion. Recognising MOs would have potentially spared approximately 1 year of delay in diagnosis.

**Conclusion:**

MOs for earlier HIV diagnosis were prevalent in Greece. Leveraging IC-guided testing and addressing barriers could support earlier diagnosis and treatment, limiting adverse health outcomes and preventing transmission.

**Key public health message**

**What did you want to address in this study?**

Late diagnosis of HIV is a persistent problem worldwide, leading to poorer health outcomes and increased transmission risks. We wanted to investigate the frequency of missed opportunities for earlier HIV testing and understand factors contributing to the delay in diagnosis in Greece.

**What have we learnt from this study?**

We found a high prevalence of missed opportunities for HIV testing in Greece, with over half of cases presenting late despite previous healthcare contacts. People of non-Greek origin had a lower chance of having a late HIV diagnosis, while higher education levels were linked to greater odds of missed opportunities. Overall, despite multiple healthcare interactions, there remains a considerable delay in diagnosis.

**What are the implications of your findings for public health?**

Our findings highlight the need to improve HIV testing based on specific health conditions that might indicate HIV infection, to address barriers to testing such as access and cultural factors, and to consider implementing routine testing for all patients. These interventions, in alignment with the 95–95–95 UNAIDS 2025 goals, are crucial for improving health outcomes and reducing HIV transmission in Greece.

## Introduction

In Western Europe, 45–50% of newly diagnosed people with HIV (PWH) are identified and enter care late, i.e. with a CD4^+^ T-cell count < 350 cells/µL or with an AIDS-defining event [1] regardless of the CD4^+^ T-cell count, with only 89% of PWH being aware of their HIV status [2-4]. Late diagnosis is associated with increased HIV-related morbidity and mortality, a suboptimal response to treatment, elevated healthcare costs and a higher transmission rate [5,6]. Late presentation remains a consistent feature of the HIV epidemic in Greece across all transmission groups [7]. Within the HIV diagnostic and treatment pillars of the 95–95–95 UNAIDS 2025 goals, there is notable room for improvement [8], particularly concerning transmission groups such as heterosexual individuals, people who inject drugs (PWID) and migrants (or PWH of non-Greek ethnicity) [9].

Identifying individuals earlier in their HIV infection course is of utmost concern. However, justification of early testing depends on the cost-effectiveness of HIV screening, which can vary based on the type, frequency, and context of the disease. Studies from the United States (US) and France suggest that routine HIV indicator condition (IC) testing is cost-effective in populations where the prevalence of undiagnosed HIV exceeds 0.1% [10,11]. Below this threshold, consideration should be given to testing only when the added cost of a test can be justified, particularly considering potential adverse consequences if HIV infection remains unidentified. Notably, cost-effectiveness analyses assume that individuals diagnosed with HIV enter care and have access to antiretroviral therapy (ART), thereby benefiting from treatment. A strategy based on IC testing can provide a reasonable alternative for enhancing HIV diagnosis in the absence of widespread pre-emptive or targeted testing coverage [12,13].

This study aimed to determine the frequency of HIV ICs preceding an HIV diagnosis at a nationwide level in Greece, using data collected from 10 infectious diseases clinics between 2019 and 2021. The objectives of this study also included: (i) delineation of the characteristics of PWH who have sought care in recent years; (ii) quantification of missed opportunities (MOs) of testing among cases with ICs; (iii) description of the nature and type of healthcare contact (HCC) that could lead to earlier diagnosis within the previous 5 years; (iv) discernment of factors associated with the likelihood of experiencing at least one MO of testing; and (v) estimation of the duration from infection to diagnosis identifying reports of missed diagnostic opportunities within this timeframe.

## Methods

### Study design and setting

This retrospective, non-interventional, multi-centre study, named MORFEAS (Missed OppoRtunities For an Early HIV diAgnosiS), included 10 infectious diseases clinics (of 16 total) across Greece. Six were located in the Athens metropolitan area (of 11 clinics) and four (of five total in other regions) Thessaloniki (Macedonia), Patras (Peloponnese), Heraklion (Crete) and Alexandroupolis (Thrace). The 10 infectious disease clinics were chosen to represent a geographically diverse sample of HIV care providers in Greece, including the major metropolitan areas of Athens and Thessaloniki where the majority of people living with HIV in the country are concentrated. These clinics also serve a socio-demographically diverse patient population and have a high volume of new HIV diagnoses per year relative to other centres in the country.

This study represents a secondary analysis of data routinely collected at the 10 HIV clinics participating in the MORFEAS study. The primary purpose of this data collection was to support the HIV mandatory surveillance system in Greece.

### Study population

#### Inclusion criteria

The study included ART-naive individuals aged ≥ 18 years diagnosed between 1 January 2019 and 31 December 2021, whose CD4^+^ T-cell count was measured within 6 months following their HIV diagnosis. The restriction to ART-naive individuals aimed to accurately classify newly diagnosed cases.

#### Exclusion criteria

Individuals diagnosed in the acute stage of HIV infection were excluded from the analysis for MOs, as these infections are considered timely detections. Acute HIV infection was defined based on meeting laboratory criteria (i.e. detection of HIV DNA or RNA by nucleic acid amplification testing (NAT) or detection of the p24 antigen in the absence of confirmed detection of HIV antibody) or having had a previous negative or indeterminate HIV test within 180 days of the first confirmed positive HIV test.

### Definition of outcomes and variables

For this study, a healthcare contact (HCC) was defined as any medical consultation or encounter (including but not limited to hospital visits, primary care consultations and blood donations) occurring either within 30 days to 5 years before the HIV diagnosis or between the date of the last negative HIV test and 30 days before the confirmed HIV diagnosis, within a maximum of 5 years before the diagnosis.

An MO of testing was defined as an HCC where an IC in the form of a clinical manifestation or laboratory abnormality was identified but did not result in an HIV test. An MO of HIV diagnosis was defined as a situation where a person who is estimated to be infected with HIV has a healthcare encounter but does not receive an HIV test.

Cases were classified as late presenters if they had a CD4^+^ T-cell count of < 350 cells/µL or an AIDS-defining condition at the time of HIV diagnosis, regardless of the CD4^+^ T-cell count.

### Data collection

To ensure a comprehensive assessment of HCCs), we conducted a thorough review of cases' medical records, including both paper-based and electronic records, covering a period of 30 days to 5 years before HIV diagnosis. Data collected included cases' sociodemographic characteristics, reasons for HIV testing, clinical status (comprising HIV disease stage [1], CD4^+^ T-cell count and plasma HIV RNA levels), hospitalisation status at the time of HIV diagnosis and vital status. This review encompassed available clinical summaries, reference letters and laboratory reports from prior medical consultations or hospitalisations to identify reported HCCs and/or any documented clinical conditions related to HIV IC.

To ensure consistency in data collection across all participating clinics, an electronic case report form containing closed-ended questions for most of the data (where applicable) was provided to each site, thereby ensuring consistency in the data collection. While our study primarily focused on hospital-based records, we also captured information about visits to private clinics. In the Greek healthcare system, HIV care is provided almost exclusively at specialised infectious disease units in public hospitals, and referrals or presentations with severe illnesses typically result in documentation of prior healthcare encounters in hospital records.

We anonymously gathered and analysed all data before the HIV diagnosis, as defined by the date of the HIV confirmatory test.

### Identifying missed opportunities for earlier diagnosis

The selection of a 5-year data collection span was based on the available data from the only Greek dataset on determinants for HIV late presentation with molecular clock-inferred HIV infection dates [14], alongside other international publications [15]. To assess HCCs within 5 years preceding the case's HIV infection diagnosis, the data were reviewed by two independent, anonymised reviewers. These reviewers determined whether each HCC should have led to HIV testing based on the presence of an IC, thus identifying MOs for earlier diagnosis.

To identify ICs, we used the ICs standardised list developed by the HIV in Europe initiative [10]. The ICs were categorised into three groups: (i) conditions that are AIDS-defining: any person not known to be HIV-positive presenting with a potentially AIDS-defining event — irrespective of the HIV prevalence in the setting where the condition is managed — should be strongly recommended for HIV testing; (ii) conditions associated with an undiagnosed HIV prevalence of > 0.1%: any person presenting with a condition associated with an undiagnosed HIV prevalence of > 0.1% should be strongly recommended for HIV testing; and (iii) conditions where not identifying the presence of HIV infection could have notable adverse implications for the individual’s clinical management despite the estimated prevalence of HIV being most likely lower than 0.1%: testing should be offered for such conditions to avoid potentially serious adverse outcomes for the individual and to maximise the potential response to the treatment of the IC [10].

### Statistical analysis

#### Statistical methods

Initially, all new diagnoses were compiled, and the prevalence of late presentation was computed from this cohort. Following this, an analysis of MOs was performed specifically on individuals with documented HCCs. Cases' demographic, clinical and epidemiological data are presented as absolute numbers and percentages for qualitative data. For quantitative data, mean values (standard deviation; SD) or median values with percentiles (25th–75th percentile) were used. The prevalence of late presentation, along with the corresponding 95% confidence interval (CI), was estimated. A comparison between the characteristics of late presenters and cases diagnosed with HIV earlier in the course of infection (non-late presenters) was conducted using the chi-square test for categorical variables and either the t-test or Mann‒Whitney U nonparametric test for continuous variables.

The frequency of MOs for receiving an earlier IC-guided HIV testing among the late presenters was described using proportions. The relationships between cases' demographic and other variables (sex, age, nationality, employment status and HIV risk category) and MOs were explored through a univariable logistic regression model. Associations were quantified using odds ratios (ORs) and their corresponding 95% CI. Additionally, a multivariable logistic regression model was used to adjust the ORs for potential confounders, and the outcomes are expressed as adjusted odds ratios (aORs) along with their 95% CI.

#### Estimation of the time of HIV seroconversion

To estimate the delay in diagnosis, we used a Bayesian method [16], which has already been applied within a study of HIV-infected migrants [17] and within the framework of HIV surveillance in Europe [18], to estimate study participants’ timing of HIV seroconversion using routinely collected clinical data at diagnosis. This integrative approach utilises a combination of subject-specific data on biomarker measurements (CD4^+^ T-cell counts and HIV viral load measurements), demographic and clinical data (indicators of AIDS onset) along with estimates from a natural history model fitted to seroconverter data from the CASCADE collaboration [19]. By comparing an individual's status at diagnosis against modelled trajectories of HIV disease progression in the absence of ART, the method provides estimates of the time gaps between likely HIV infection and diagnosis dates.

All the statistical analyses were performed using Stata 17.0 (StataCorp). A p value of < 0.05 was considered statistically significant.

## Results

The study population consisted of 823 treatment-naive individuals diagnosed with HIV over a 3-year period (2019–21) from the 10 participating infectious diseases clinics, representing more than 44.2% (823/1,864) of the newly reported cases at a national level. The median age was 37.6 (25th–75th percentile: 29.6–45.3) years. The majority of participants were male (n = 710; 86.3% vs n = 113; 13.7% female), of Greek origin (n = 612; 74.4%), living in urban areas at the time of diagnosis (n = 703; 85.4%) and had full-time employment (n = 367; 44.6%). A comparative analysis of demographic, clinical and HIV-related characteristics among the total sample (n = 823) and participants with available healthcare contact information (n = 517) and those without (n = 306) is provided in Supplementary Table S1.

Of 710 male participants, 66.9% (n = 475) were men who have sex with men (MSM), while 23.3% (n = 192) had acquired HIV through heterosexual transmission (13.1%; 93/710 males and 88.4%; 99/112 females). Additionally, 12.0% (99/823) of participants were PWID (12.5%; 89/710 males and 8.9%; 10/112 females) and 6.9% (57/823) had an unknown mode of transmission. Among the 823 cases, only 323 (39.2%) reported previous HIV testing, and 428 (52.0%; 95% CI: 48.6–55.4) were late presenters (clinical AIDS or CD4^+^ T-cell count < 350 cells/μL), with a median CD4^+^ T-cell count at diagnosis of 338 (25th–75th percentile: 167–516) cells/μL (refer to Supplementary Table S1).Fifty-four individuals were diagnosed with acute HIV infection and were excluded from further analysis. Of the 823 cases, 71.2% (n = 586) were diagnosed as asymptomatic (CDC stage A [1]), while 14.9% (n = 123) had already developed AIDS at diagnosis. The three most frequently reported AIDS-defining conditions were *Pneumocystis jirovecii* pneumonia (30.9%), wasting syndrome (22.8%) and Kaposi’s sarcoma (18.7%).

### Characteristics of the study population with available information on healthcare contacts

**Figure 1:**
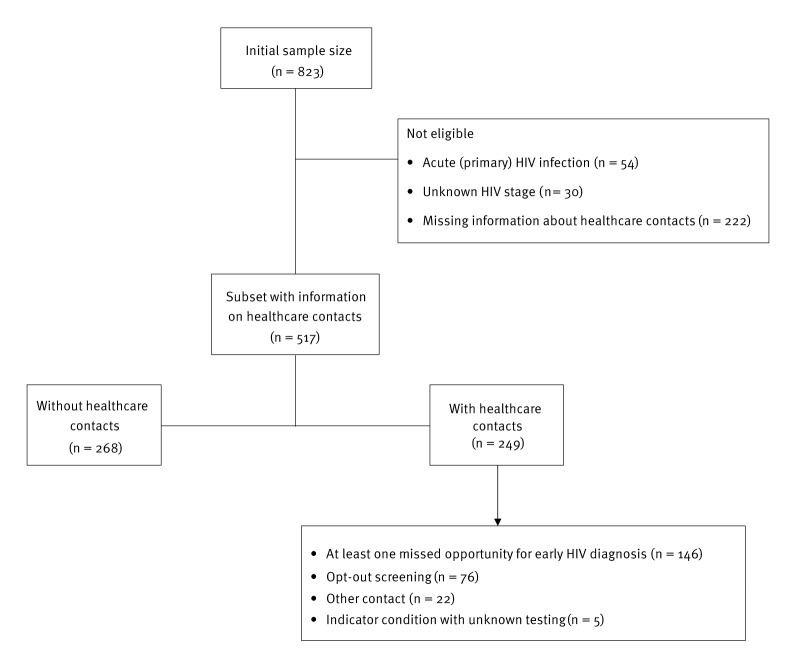
Flowchart of participant enrolment and inclusion in the MORFEAS study, Greece, 2019–2021 (n = 823)

In a subset of 517 individuals with available information on HCCs (Figure 1, Table 1), 48.2% (n = 249) had at least one HCC. Among those, 55.0% (n = 137) were MSM, while 27.7% (n = 69) were heterosexual. Of those individuals with an HCC, 59.0% (n = 147) were late presenters, with a median (25th–75th percentile) CD4^+^ T-cell count at diagnosis of 287 (120–466) cells/μL, and 18.1% (n = 45) were diagnosed at stage C (Table 1).

**Table 1:** Characteristics of participants at first clinic visit after HIV diagnosis, stratified by healthcare contacts, Greece, 2019–2021 (n = 517)

Characteristics		Participants (n = 517)		Healthcare contacts				p value
				No (n = 268)		Yes (n = 249)		
		n	%	n	%	n	%	
Sex								
Male		447	86.5	237	88.4	210	84.3	0.155^a^
Female		69	13.3	30	11.2	39	15.7	
Other		1	0.2	1	0.4	0	0.0	
Age in years								
Mean (SD)		39.2 (12.3)		37.4 (11.1)		41.1 (13.3)		< 0.001^b^
Median (25^th^–75^th^ percentile)		37.6 (29.6–46.3)		37.2 (28.3–44.9)		38.5 (31.6–48.1)		0.004^c^
Birth country								
Greece		420	81.2	212	79.1	208	83.5	0.200^d^
Other		97	18.8	56	20.9	41	16.5	
Place of residence								
Urban centre (> 10,000 inhabitants)		462	89.4	242	90.3	220	88.4	0.840^a^
Semi-urban area (2,000–10,000 inhabitants)		34	6.6	17	6.3	17	6.8	
Rural area (< 2,000 inhabitants)		17	3.3	8	3.0	9	3.6	
Unknown		4	0.8	1	0.4	3	1.2	
Education level								
Up to lower secondary education		56	10.8	20	7.5	36	14.5	< 0.001^d^
Upper secondary education up to post-secondary non-tertiary education		171	33.1	96	35.8	75	30.1	
Bachelor's degree or higher		174	33.7	109	40.7	65	26.1	
Unknown		116	22.4	43	16.0	73	29.3	
Occupational status								
Full-time		264	51.1	165	61.6	99	39.8	< 0.001^d^
Part-time		58	11.2	27	10.1	31	12.4	
Unemployed		110	21.3	49	18.3	61	24.5	
Other		35	6.8	10	3.7	25	10.0	
Unknown		50	9.7	17	6.3	33	13.3	
Year of diagnosis								
2019		183	35.4	92	34.3	91	36.5	0.870^d^
2020		175	33.8	92	34.3	83	33.3	
2021		159	30.8	84	31.3	75	30.1	
HIV testing within 5 years prior to diagnosis								
Yes		236	45.6	158	59.0	78	31.3	< 0.001^d^
No		163	31.5	69	25.7	94	37.8	
Unknown		118	22.8	41	15.3	77	30.9	
Initiation of antiretroviral therapy								
Yes		513	99.2	265	98.9	248	99.6	0.350^d^
No		4	0.8	3	1.1	1	0.4	
Transmission risk group								
MSM		325	62.9	188	70.1	137	55.0	0.005^d^
PWID		44	8.5	18	6.7	26	10.4	
Heterosexual		120	23.2	51	19.0	69	27.7	
Unspecified		28	5.4	11	4.1	17	6.8	
Hospitalisation at HIV diagnosis								
Yes		103	19.9	33	12.3	70	28.1	< 0.001^d^
No		414	80.1	235	87.7	179	71.9	
Stage of HIV infection [1]								
Α		391	75.6	223	83.2	168	67.5	< 0.001^d^
Β		55	10.6	19	7.1	36	14.5	
C		71	13.7	26	9.7	45	18.1	
Available viral load								
No		169	32.7	91	34.0	78	31.3	0.520^d^
Yes		348	67.3	177	66.0	171	68.7	
Viral load (log_10_ copies/ml)								
Mean (SD)		4.7	1.2	4.7	1.1	4.6	1.2	0.730^b^
Median (25^th^–75^th^ percentile)		4.8	4.0–5.4	4.8	4.0–5.3	4.8	3.9–5.5	0.860^c^
CD4^+^ T-cell count (cells/μL)								
Mean (SD)		375 (272.0)		423 (275.0)		324 (259.0)		< 0.001^b^
Median (25^th^–75^th^ percentile)		356 (164–531)		420 (221–582)		287 (120–466)		< 0.001^c^
Distribution	< 350^d^	252	49.0	105	39.5	147	59.3	< 0.001^e^
	350–500	109	21.2	63	23.7	46	18.5	
	≥ 500	153	29.8	98	36.8	55	22.2	
Late presenters^f^								
No		264	51.1	162	60.4	102	41.0	< 0.001^d^
Yes		253	48.9	106	39.6	147	59.0	
Death								
Yes		15	2.9	6	2.2	9	3.6	0.190^d^
No		482	93.2	255	95.1	227	91.2	
Unknown		20	3.9	7	2.6	13	5.2	

MSM: men who have sex with men; PWID: people who inject drugs; SD: standard deviation.

^a^ Fisher's exact test.

^b^ Student’s t-test.

^c^ Mann-Whitney U test.

^d^ Two participants with CD4^+^ T-cell count ≥ 350 cells/μL were classified as late presenters because of AIDS-defining illnesses at diagnosis.

^e^ Pearson's chi-square test.

^f^ Defined as clinical AIDS or CD4^+^ T-cell count < 350 cells/μL.

For statistical tests, a p value < 0.05 was considered significant.

### Frequency and characteristics of missed opportunities for HIV testing

**Figure 2:**
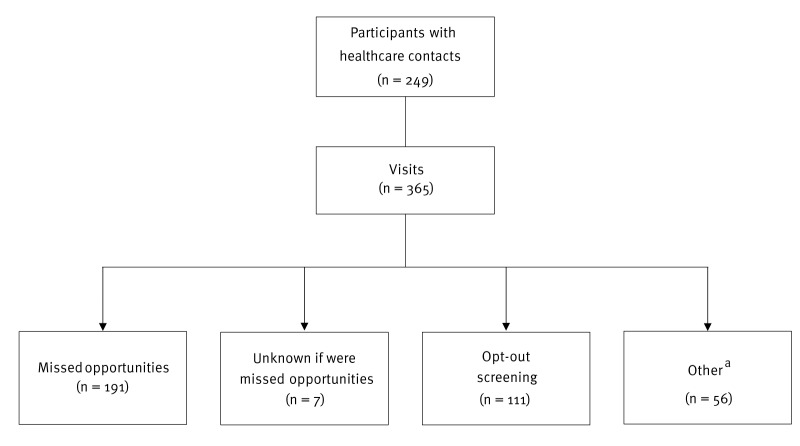
Types of visits to the healthcare system among participants who had at least one missed opportunity for earlier HIV diagnosis, Greece, 2019–2021 (n = 365)

^a^ Neither missed opportunities nor opt-out screening.

Overall, the 249 cases with HCC had a total of 365 visits to various healthcare settings during the 5 years preceding HIV diagnosis. Among these, 191 visits (52.3%; 191/365), involving 146 cases, were MOs for testing because the cases had an IC for HIV, yet the test was not offered (Figure 2). The most prominent ICs were related to the diagnosis of sexually transmitted infections (STIs) (39.8%), fever (11.0%), community-acquired pneumonia (6.8%), herpes zoster (5.2%) or unexplained weight loss (4.7%) (Table 2). More than 81.2% (155/191) of the MOs of testing were due to conditions associated with an undiagnosed HIV prevalence greater than 0.1% (Table 2).

**Table 2:** Distribution of HIV indicator conditions of 146 participants who had at least one missed opportunity for HIV testing, Greece, 2019–2021 (n = 191 visits)

Characteristics	Total visits (n = 191)	
	n	%
Category of missed opportunity		
Conditions which are AIDS-defining among PWH	6	3.1
Conditions associated with an undiagnosed HIV prevalence of > 0.1%^a^	155	81.2
Other conditions considered likely to have an undiagnosed HIV prevalence of > 0.1%^b^	18	9.4
Conditions where not identifying the presence of HIV infection may have notable adverse implications for the individual’s clinical management despite that the estimated prevalence of HIV is most likely lower than 0.1%	2	1.0
Opioid substitution treatment	10	5.2
Indicator condition		
Sexually transmitted infections	76	39.8
Unexplained fever	21	11.0
Community-acquired pneumonia	13	6.8
Herpes zoster	10	5.2
Opioid substitution treatment	10	5.2
Unexplained weight loss	9	4.7
Hepatitis B or C (acute or chronic)	8	4.2
Mononucleosis-like illness	8	4.2
Pregnancy (implications for the unborn child)	7	3.7
Unexplained leukocytopenia/thrombocytopenia lasting > 4 weeks	3	1.6
Seborrheic dermatitis/exanthema	3	1.6
Anal cancer/dysplasia	3	1.6
Unexplained lymphadenopathy	3	1.6
Unexplained chronic diarrhoea	3	1.6
Hepatitis A	3	1.6
Candidiasis, oesophageal	2	1.0
Multiple sclerosis-like disease	2	1.0
Cancer	2	1.0
Cytomegalovirus, other (except liver, spleen, glands)	1	0.5
*Pneumocystis jirovecii* pneumonia	1	0.5
Non-Hodgkin lymphoma	1	0.5
Pneumonia, recurrent (2 or more episodes in 12 months)	1	0.5
Unexplained chronic renal impairment	1	0.5
Place of healthcare contact		
Private clinic	52	27.2
Private hospital	4	2.1
Primary healthcare settings	2	1.0
Secondary healthcare settings	21	11.0
Tertiary healthcare settings	27	14.1
Unknown	85	44.5
Medical specialty		
Internist/general practitioner/specialist internist	48	25.1
Dermatologist	17	8.9
Gastroenterologist	9	4.7
Pulmonologist	7	3.7
Urologist	4	2.1
Gynaecologist	3	1.6
Other	15	7.9
Unknown	88	46.1
Year of contact with the healthcare system		
2014–16	19	9.9
2017	20	10.5
2018	50	26.2
2019	50	26.2
2020–21	52	27.2

PWH: people with HIV.

^a^ Conditions associated with an undiagnosed HIV prevalence of > 0.1% refers to the list of HIV indicator conditions confirmed by the HIV Indicator Diseases Across Europe Study (HIDES) to have an undiagnosed HIV prevalence exceeding 0.1% [11].

^b^ Other conditions considered likely to have an undiagnosed HIV prevalence of > 0.1% includes additional conditions that, based on more recent evidence or expert clinical judgment, are believed to meet the > 0.1% prevalence threshold, but have not been formally validated in the HIDES study.

### Healthcare settings and providers associated with missed opportunities

Private clinics accounted for 27.2% of the MOs of testing, followed by tertiary and secondary healthcare settings, with 14.1% and 11.0%, respectively. The medical specialty most commonly associated with MOs of testing was internists/general practitioners (25.1%) (Table 2).

### Predictors associated with the likelihood of experiencing at least one missed opportunity for earlier HIV testing

Table 3 presents the results of univariable and multivariable analyses examining the risk factors associated with MOs of HIV testing. In the univariable analysis, factors significantly associated with lower odds of MOs for HIV testing included being born in a country other than Greece, receiving an HIV diagnosis through routine screening during blood donation and having heterosexual transmission risk (compared with MSM). Individuals with a higher education level (Bachelor's degree or higher) had higher odds.

**Table 3:** Determinants of missed opportunities for early HIV diagnosis among individuals with prior healthcare contact, Greece, 2019–2021 (n = 244^a^)

Variable	Univariable		p value	Multivariable		p value
	OR	95% CI		aOR	95% CI	
Sex						
Male	Ref.			Ref.		
Female	0.62	0.31–1.25	0.181	0.91	0.42–1.97	0.817
Age at HIV diagnosis						
Per 1-year increase	1.01	0.99–1.03	0.451	1.00	0.98–1.02	0.778
Birth country						
Greece	Ref.			Ref.		
Other	0.46	0.23–0.90	0.025	0.48	0.22–1.02	0.055
Place of residence						
Urban centre (> 10,000 inhabitants)	Ref.			NI		
Semi-urban area (2,000 < inhabitants < 10,000)	0.69	0.26–1.87	0.469			
Rural area (< 2,000 inhabitants)	0.49	0.13–1.89	0.302			
Education level						
Up to upper secondary education or post-secondary non-tertiary education	Ref.			Ref.		
Bachelor's degree or higher	2.29	1.18–4.44	0.014	2.31	1.15–4.64	0.019
Unknown	1.33	0.73–2.43	0.359	1.46	0.77–2.73	0.244
Occupational status						
Full time	Ref.			NI		
Part time	0.53	0.23–1.22	0.138			
Unemployed	0.88	0.46–1.70	0.700			
Other	1.08	0.43–2.71	0.862			
Unknown	1.07	0.47–2.43	0.876			
Year of diagnosis						
2019	Ref.			NI		
2020	1.29	0.70–2.38	0.414			
2021	1.36	0.72–2.56	0.336			
HIV testing						
Yes	Ref.			NI		
No	0.56	0.30–1.06	0.073			
Unknown	0.62	0.32–1.21	0.160			
Transmission risk group						
MSM	Ref.			NI		
PWID	1.19	0.48–2.94	0.707			
Heterosexual	0.44	0.24–0.80	0.007			
Unspecified	0.76	0.27–2.12	0.593			
HIV diagnosis through blood donation screening						
No	Ref.			Ref.		
Yes	0.1	0.01–0.88	0.038	0.08	0.01–0.71	0.023
Unknown	0.31	0.03–3.50	0.345	0.32	0.03–3.92	0.373
Hospitalisation at HIV diagnosis						
Yes	Ref.			NI		
No	0.97	0.55–1.72	0.928			
Stage of HIV Infection [1]						
Α	Ref.			NI		
Β	0.75	0.36–1.56	0.447			
C	1.84	0.88–3.83	0.104			
Late presenters^b^						
No	Ref.			NI		
Yes	1.45	0.86–2.43	0.164			
Death						
Yes	Ref.			NI		
No	0.89	0.21–3.80	0.871			
Unknown	0.96	0.16–5.90	0.965			

aOR: adjusted odds ratio; CI: confidence interval; NI: not included; Ref.: reference category.

^a^ Of the 249 healthcare contacts, five participants had seven visits where missed opportunity status was unknown.

^b^ Defined as clinical AIDS or CD4^+^ T-cell count < 350 cells/μL.

Upon adjustment, individuals born in countries other than Greece were less likely to experience an MO of testing, although this association was marginally significant (aOR: 0.48; 95% CI: 0.22–1.02; p = 0.055). Additionally, HIV testing in conjunction with blood donation was associated with lower odds compared with other reasons for HIV testing that resulted in an HIV diagnosis (OR: 0.08; 95% CI: 0.01–0.71; p = 0.023). Individuals at higher risk to experience an MO of testing were those with higher education levels compared with those with up to upper secondary education or postsecondary nontertiary education (OR: 2.31; 95% CI: 1.15–4.64; p = 0.019). Age, sex, place of residence, occupational status and year of diagnosis were not associated with the probability of experiencing an MO of HIV testing (Table 3).

### Estimation of HIV seroconversion time

After estimating the HIV seroconversion time of each study participant, the median time gap between seroconversion and diagnosis for the total sample of 823 individuals was 3.2 years (25th–75th percentile: 1.0–5.7; 95% CI: 2.7–3.7). Among the 249 individuals with at least one HCC, the estimated time gap between seroconversion and diagnosis was 3.7 years (25th–75th percentile: 1.9–6.3; 95% CI: 3.2–4.2) (Table 4). Of these 249 individuals, 124 (49.8%) had at least one HCC after their estimated date of seroconversion but before the date of HIV diagnosis (MO for HIV diagnosis). Among the 249 cases, approximately half of the total number of visits (43.8%) occurred after the estimated date of seroconversion. The median time between the earliest MO and diagnosis was 0.9 (25th–75th percentile: 0.4–2.2) years. The timeline visualisation of these key periods in HIV diagnosis progression is provided in Supplementary Figure S1.

**Table 4:** Estimated time from HIV seroconversion to diagnosis, stratified by healthcare contacts and missed opportunities, Greece, 2019–2021 (n = 823)

Category	n	Time from seroconversion to diagnosis		
		Median years	25th–75th percentile	95% CI of median
Total sample size	823	3.2	1.0–5.7	2.7–3.7
Subset with information on healthcare contacts	517	3.0	0.8–5.1	2.5–3.5
Without healthcare contacts	268	1.9	0.4–4.2	1.4–2.4
With healthcare contacts	249	3.7	1.9–6.3	3.2–4.2
At least one missed opportunity for early HIV diagnosis	146	3.8	1.8–6.5	3.2–4.3

CI: confidence interval.

## Discussion

Late HIV diagnosis remains a persistent challenge across most European countries. About half (50.6%) of those diagnosed in 2022 were classified as late presenters [3], emphasising the urgent need to explore the underlying obstacles hindering timely access to testing and care. This is the first study to our knowledge that addresses MOs for earlier diagnosis at a national level in Greece. Unlike a previous single-centre study in Greece that focused on the period 2003–16 [20], our nationwide study provides updated estimates of MOs in the context of the evolving Greek HIV epidemic, while describing in detail the clinical profile of newly diagnosed PWH, including late presenters. In addition, it provides an estimation of the time delay between infection and diagnosis, offering a clearer understanding of MOs timing vs using arbitrary time limits.

Since the reporting of MOs in a single-centre study in 2016 [20], the Greek HIV epidemic has undergone notable changes, notably with an increased frequency of new cases among non-Greek ethnicities and heterosexual individuals [7]. The incidence of HIV among PWID has remained stable since the original HIV outbreak in this population approximately a decade ago in Athens, while another one has emerged in an urban area beyond Athens during the COVID-19 pandemic [21,22]. In this nationwide representative sample in Greece for the years 2019–21, an MO for earlier diagnosis after the estimated infection date was identified for ca 50% of PWH with available HCC information. If testing had been performed at the MO, the time from seroconversion to diagnosis could have been decreased by ca 1 year. In 52.3% of the visits with an indication to test for HIV, healthcare providers did not offer the test.

To address this issue, IC-guided testing has been developed as a targeted strategy by a pan-European initiative to facilitate early HIV diagnosis [11,23]. During the COVID-19 pandemic, this strategy became notably important because of restricted access to checkpoints and the absence of a formal framework for HIV self-testing. The strategy includes a variety of medical conditions that, when identified, signal an HIV prevalence of > 0.1% and warrant HIV testing. However, a meta-analysis published in 2021, covering studies conducted between 2009 and 2020, revealed considerable variation in HIV test ratios per IC in Western countries [24]. Despite the existing evidence, an implementation gap persists between European and national guidelines concerning HIV testing. A systematic review of HIV testing guidelines on the presence of ICs across Europe revealed notable variability in the incorporation of IC-based testing. While 69% of national guidelines acknowledged associations between certain clinical conditions and HIV, only 46% of them explicitly recommending HIV testing in the presence of these conditions. Even more concerning, only 42% recommended HIV testing for AIDS-defining conditions [25]. This inconsistency is also evident in Greece. A recent study examining specialty guidelines in the country found that while 64% of guidelines for AIDS-defining conditions and 22% of guidelines for ICs mentioned an association with HIV, appropriate HIV testing was recommended in only 18% of AIDS-defining conditions guidelines and 28% of ICs guidelines [26]. These findings highlight a persistent gap between recognising HIV-associated conditions and translating that recognition into clear testing recommendations, both at the European level and within Greece specifically.

This study revealed that 81.2% of MOs were associated with conditions with undiagnosed HIV prevalence exceeding 0.1%. Studies on MOs for earlier HIV diagnosis have shown that individuals with undiagnosed HIV often visit healthcare settings multiple times in the years preceding their diagnosis [27], with visits occurring not only in emergency departments but also in secondary care settings [28]. The estimation of MOs can vary based on the definitions used and the time frame selected before HIV diagnosis (e.g. 1 year vs 5 years). In this study, we estimated the time between the earliest MO and diagnosis, which was 0.9 years.

Notably, in our study, individuals with higher education levels exhibited a greater likelihood of having an MO for HIV testing, whereas those born outside Greece were less likely to encounter such opportunities. Misconceptions regarding HIV exposure influence testing rates, while stigma and stereotyping by healthcare providers may impede testing for those in need [29]. Anticipated HIV stigma can deter people, particularly among key populations, contributing to lower testing rates [30]. Conversely, individuals whose HIV was initially diagnosed through blood donation screening were associated with a lower risk of encountering an MO for earlier diagnosis. Individuals who wish to test for HIV may attempt ‘stigma management techniques’ to avoid the potential stigmatisation associated with testing; for example, test for HIV by receiving other non-stigmatised services that include an HIV test, such as donating blood. It remains crucial to normalise HIV testing and to provide access to non-judgmental, stigma-free testing options in traditional and nontraditional settings, including home-based self-testing, potentially improving earlier diagnosis [31]. Improving testing accessibility in healthcare settings, mitigating discrimination and stigma through healthcare provider training on HIV testing guidelines, removing barriers, and introducing free at-home testing and opt-out HIV testing in emergency departments are pivotal strategies for reducing late diagnosis rates [32]. Indicator condition-guided testing for HIV remains insufficiently practiced compared with opt-out testing strategies [24]. In this study, we found that the median time between the earliest MO and diagnosis was 0.9 years, confirming that IC-guided testing does not align with best practices. Effectively combating the HIV epidemic hinges on pre-emptive testing and promoting indication-based testing, emphasising the importance of communicating ICs. Still, of note, HIV testing guided by IC may have inherent limitations in diagnosing HIV infection at an earlier stage.

Our study highlights the notable delay between HIV seroconversion and diagnosis in Greece during 2019–21, with more than half of the participants presenting late. Among PWH with identifiable testing opportunities, with a notable portion experiencing at least one MO for HIV testing post-seroconversion. These findings suggest that addressing MOs for HIV testing could potentially lead to earlier diagnoses, though the exact time gained is subject to uncertainty and requires further investigation. Leveraging IC-guided testing and addressing barriers to testing could support earlier diagnosis and treatment, potentially limiting adverse health outcomes and preventing transmission. Expanding opt-out testing to all healthcare settings could improve access to testing, reduce stigma, and help to identify undiagnosed HIV infections earlier.

This study provides the first nationally representative data on MOs for HIV diagnosis in Greece, offering insights into a healthcare system experiencing evolving epidemic dynamics. By integrating MO assessment with seroconversion timing estimation, we quantify actual time lost between infection and diagnosis. Our analysis reveals specific risk factors for MOs within the Greek context and identifies key areas within the healthcare system where interventions could be targeted. These findings have important implications for improving HIV testing strategies and reducing late diagnoses in Greece and potentially in other European countries with similar healthcare systems.

This study has several strengths. The sample encompasses diverse geographical regions across Greece and includes a substantial proportion of recent diagnoses. This diverse sample, combined with the nationwide distribution of infectious diseases clinics, provided sufficient representation across all transmission groups for analysis. Moreover, meticulous and standardised recording of medical history, along with the involvement of two anonymised reviewers, allowed the study to establish structured criteria for defining and identifying MOs for earlier HIV diagnosis. This approach enabled a detailed assessment of this critical issue. Additionally, the Bayesian method used enabled the estimation of infection timing with the highest accuracy. The study's clinical profiling and healthcare system analysis yielded detailed insights into patient-provider interactions and system-level barriers. Finally, these findings carry important policy implications, providing an evidence base for improving early HIV detection strategies within the Greek healthcare system.

However, inherent limitations are present. Firstly, as a retrospective study reliant on existing medical records, it is susceptible to potential inaccuracies and incompleteness in the data. The completeness of HCCs ascertainment is a key limitation. While we are confident that our rigorous methodology captured the vast majority of HCCs documented in medical records, we cannot exclude the possibility that some encounters went unrecorded. Greece does not have a national integrated health record system, which hinders the ability to comprehensively track patients' interactions with different healthcare providers. Secondly, the available data concerning HCCs were limited to 517 individuals out of the initial 823 participants, potential introduce selection bias. Participants with available HCC information were more likely to be born in Greece, reside in urban areas, have higher education levels, be employed full-time, have undergone recent HIV testing and have initiated antiretroviral therapy. They also had less advanced HIV disease progression and a lower percentage of deaths. These differences suggest that our sample with HCC information may represent a group with better access to healthcare and potentially more health-seeking behaviours, possibly leading to an underestimation of MOs in the overall HIV-positive population in Greece, particularly among those less engaged with the healthcare system. Thirdly, another key limitation is the inherent uncertainty in estimating the date of HIV infection. While we employed a robust Bayesian method to estimate the timing of seroconversion [16], it is important to acknowledge that these estimates are subject to variability. Finally, while our study offers valuable insights into MOs for HIV diagnosis in Greece, the generalisability of our findings may be limited. Our focus on specific infectious disease clinics and the characteristics of our sample may not fully represent the broader Greek population or be directly applicable to other healthcare systems. Future research encompassing a wider range of healthcare settings and patient populations would be valuable to confirm the broader applicability of our findings.

## Conclusions

Our findings demonstrate critical gaps in HIV testing implementation in Greece, and the need for changes in healthcare delivery to enable earlier diagnosis. By addressing missed opportunities through expanded opt-out testing, reducing stigma, and strengthening indicator condition-guided testing across healthcare settings, we can improve timely access to HIV care and treatment. These targeted interventions have the potential to significantly impact both individual health outcomes and public health efforts to control HIV transmission in Greece and similar healthcare settings.

## Supplementary Data

292000 Supplement
