# Supplementary material for: Missed opportunities for early HIV diagnosis in Greece: The MORFEAS study, 2019 to 2021
Source: Euro Surveill. 2024 Nov 28;29(48):2400138. doi: 10.2807/1560-7917.ES.2024.29.48.2400138 (PMC11605803; doi:10.2807/1560-7917.ES.2024.29.48.2400138)
Supplement: Supplement [file 24-00138_PSICHOGIOU_Supplement.pdf]

Supplementary Materials

Disclaimer: This supplementary material is hosted by *Eurosurveillance* as supporting information alongside the article **"Missed OppoRtunities For an Early HIV diAgnosis in Greece: The MORFEAS study, 2019-2021"**, on behalf of the authors, who remain responsible for the accuracy and appropriateness of the content. The same standards for ethics, copyright, attributions and permissions as for the article apply. Supplements are not edited by *Eurosurveillance* and the journal is not responsible for the maintenance of any links or email addresses provided therein.

Supplementary Table S1. Characteristics of participants at their first visit to the infectious disease unit/outpatient clinic, among those with available information on healthcare contacts and the remaining initial sample.

	**Total sample N = 823**		**The rest of sample N = 306**		**With healthcare contacts information N = 517**		**p-value**
Sex, n (%)							0.900
Male	710	86.3	263	85.9	447	86.5	
Female	112	13.6	43	14.1	69	13.3	
Other	1	0.1	0	0.0	1	0.2	
Age at 1st visit in hospital (years)							
mean (sd)	38.5	11.6	37.5	10.3	39.2	12.3	0.044
median (25th-75th percentile)	37.6	29.6-45.3	37.3	29.7-44.1	37.6	29.6-46.3	0.200
Birth country, n (%)							<0.001
Greece	612	74.4	192	62.7	420	81.2	
Other	183	22.2	86	28.1	97	18.8	
Unknown	28	3.4	28	9.2	0	0.0	
Place of residence, n (%)							<0.001
Urban center (>10,000 inhabitants)	703	85.4	241	78.8	462	89.4	
Semi-urban area (2,000<inhabitants<10,000)	52	6.3	18	5.9	34	6.6	
Rural area (<2,000 inhabitants)	30	3.6	13	4.2	17	3.3	
Unknown	38	4.6	34	11.1	4	0.8	
Education level, n (%)							<0.001
Up to lower secondary education	66	8.0	10	3.3	56	10.8	
Upper secondary education up to post-secondary non-tertiary education	229	27.8	58	19.0	171	33.1	
Bachelor's degree or higher	227	27.6	53	17.3	174	33.7	
Unknown	301	36.6	185	60.5	116	22.4	
Occupational status, n (%)							<0.001
Full time	367	44.6	103	33.7	264	51.1	
Part time	72	8.7	14	4.6	58	11.2	
Unemployed	197	23.9	87	28.4	110	21.3	
Other	41	5.0	6	2.0	35	6.8	
Unknown	146	17.7	96	31.4	50	9.7	
Year of diagnosis, n (%)							0.770

2019	291	35.4	108	35.5	183	35.4	
2020	284	34.6	109	35.9	175	33.8	
2021	246	30.0	87	28.6	159	30.8	
HIV testing, n (%)							<0.001
Yes	323	39.2	87	28.4	236	45.6	
No	204	24.8	41	13.4	163	31.5	
Unknown	296	36.0	178	58.2	118	22.8	
Initiation of Antiretroviral Therapy, n (%)							<0.001
Yes	785	95.4	272	88.9	513	99.2	
No	9	1.1	5	1.6	4	0.8	
Unknown	29	3.5	29	9.5	0	0.0	
Transmission risk group, n (%)							<0.001
MSM	475	57.7	150	49.0	325	62.9	
PWID	99	12.0	55	18.0	44	8.5	
HETERO	192	23.3	72	23.5	120	23.2	
Unspecified	57	6.9	29	9.5	28	5.4	
Hospitalization at HIV diagnosis, n (%)							<0.001
Yes	199	24.2	96	31.4	103	19.9	
No	594	72.2	180	58.8	414	80.1	
Unknown	30	3.6	30	9.8	0	0.0	
Stage of HIV Infection, n (%)							<0.001
A	586	71.2	195	63.7	391	75.6	
B	84	10.2	29	9.5	55	10.6	
C	123	14.9	52	17.0	71	13.7	
Unknown	30	3.6	30	9.8	0	0.0	
Available viral load, n (%)							0.061
No	250	30.4	81	26.5	169	32.7	
Yes	573	69.6	225	73.5	348	67.3	
Viral load (log_{10} copies/ml)							
mean (sd)	4.7	1.3	4.8	1.4	4.7	1.2	0.360
median (25-75th percentile)	4.8	4.0-5.5	4.8	4.0-5.7	4.8	4.0-5.4	0.290
$CD4_+$ count (cells/μL)							
mean (sd)	360	262.0	335	244.0	375	272.0	0.036
median (25-75th percentile)	338	167-516	315	174-470	356	164-531	0.038
Distribution							0.063
<350	426	52.0	174	57.0	252	49.0	
[350, 500)	169	20.6	60	19.7	109	21.2	
≥ 500	224	27.4	71	23.3	153	29.8	
Late presenters, n (%)							0.022
No	395	48.0	131	42.8	264	51.1	
Yes	428	52.0	175	57.2	253	48.9	
Death, n (%)							<0.001
Yes	27	3.3	12	3.9	15	2.9	
No	707	85.9	225	73.5	482	93.2	
Unknown	89	10.8	69	22.5	20	3.9	

Supplementary Figure S1. Median time between estimated seroconversion and HIV diagnosis and between estimated seroconversion and the first missed opportunity preceding HIV diagnosis.

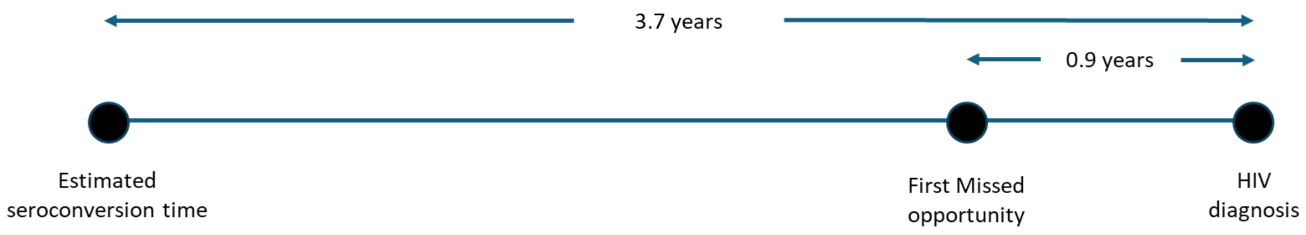

Results

Among the 823 participants included in the full sample, 517 (62.8%) had accessible healthcare contact information. This subgroup, in contrast to the remaining 306, exhibited a greater likelihood of being born in Greece (81.2% vs 62.7%, p < 0.001), residing in urban areas (89.4% vs 78.8%, p < 0.001), holding a bachelor's degree or higher (33.7% vs 17.3%, p < 0.001), being employed full-time (51.1% vs 33.7%, p <0.001), having undergone an HIV test within 5 years of diagnosis (45.6% vs 28.4%, p < 0.001), and initiating antiretroviral therapy (99.2% vs 88.9%, p < 0.001).

The mean age at the first hospital visit for those with available contact data was 39.2 years, compared to 37.5 years for the remaining participants (p = 0.044). Among all participants, 57.7% were identified as men who have sex with men (MSM), with the proportion rising to 62.9% among those with contact information. Hospitalization at HIV diagnosis was lower among participants with contact data than among the remaining individuals (19.9% vs 31.4%, p < 0.001). Late presentation was less frequent among those with contact data (48.9%) than among the remaining participants (57.2%, p = 0.022). The prevalence of death was lower among participants with contact data (2.9%) than among the other participants (3.9%) (p < 0.001).

In summary, participants with available healthcare contact data tended to reside more frequently in urban areas, attain higher education levels, and engage more actively in HIV treatment than did the remaining participants. Additionally, those with contact data had less advanced HIV disease progression and a lower percentage of deaths.
